# A comparison of health expectancies over two decades in England: results of the Cognitive Function and Ageing Study I and II

**DOI:** 10.1016/S0140-6736(15)00947-2

**Published:** 2016-02-20

**Authors:** Carol Jagger, Fiona E Matthews, Pia Wohland, Tony Fouweather, Blossom C M Stephan, Louise Robinson, Antony Arthur, Carol Brayne, Medical Research Council Cognitive Function and Ageing Collaboration

**Affiliations:** a Institute of Health and Society, Faculty of Medicine, Newcastle University, Newcastle, UK; b Newcastle University Institute for Ageing, Newcastle University, Newcastle, UK; c Medical Research Council (MRC) Biostatistics Unit, Cambridge Institute of Public Health, Cambridge, UK; d School of Health Sciences, University of East Anglia, Norwich, UK; e Department of Public Health and Primary Care, Cambridge Institute of Public Health, Cambridge University, Cambridge, UK

## Abstract

**Background:**

Whether rises in life expectancy are increases in good-quality years is of profound importance worldwide, with population ageing. We investigate how various health expectancies have changed in England between 1991 and 2011, with identical study design and methods in each decade.

**Methods:**

Baseline data from the Cognitive Function and Ageing Studies in populations aged 65 years or older in three geographically defined centres in England (Cambridgeshire, Newcastle, and Nottingham) provided prevalence estimates for three health measures: self-perceived health (defined as excellent–good, fair, or poor); cognitive impairment (defined as moderate–severe, mild, or none, as assessed by Mini-Mental State Examination score); and disability in activities of daily living (defined as none, mild, or moderate–severe). Health expectancies for the three regions combined were calculated by the Sullivan method, which applies the age-specific and sex-specific prevalence of the health measure to a standard life table for the same period.

**Findings:**

Between 1991 and 2011, gains in life expectancy at age 65 years (4·5 years for men and 3·6 years for women) were accompanied by equivalent gains in years free of any cognitive impairment (4·2 years [95% CI 4·2–4·3] for men and 4·4 years [4·3–4·5] for women) and decreased years with mild or moderate–severe cognitive impairment. Gains were also identified in years in excellent or good self-perceived health (3·8 years [95% CI 3·5–4·1] for men and 3·1 years [2·7–3·4] for women). Gains in disability-free years were much smaller than those in excellent–good self-perceived health or those free from cognitive impairment, especially for women (0·5 years [0·2–0·9] compared with 2·6 years [2·3–2·9] for men), mostly because of increased mild disability.

**Interpretation:**

During the past two decades in England, we report an absolute compression (ie, reduction) of cognitive impairment, a relative compression of self-perceived health (ie, proportion of life spent healthy is increasing), and dynamic equilibrium of disability (ie, less severe disability is increasing but more severe disability is not). Reasons for these patterns are unknown but might include increasing obesity during previous decades. Our findings have wide-ranging implications for health services and for extension of working life.

**Funding:**

UK Medical Research Council.

## Introduction

In most developed countries worldwide, life expectancy is increasing by at least 2 years every decade and does not seem to be slowing down, at least for life expectancy at age 60 years.^1^ Nevertheless, disability trends have not shown such clear improvement, with results of studies in the USA and Europe showing increases, decreases, or stagnation in disability prevalence over time.^2^, ^3^, ^4^, ^5^, ^6^, ^7^, ^8^, ^9^, ^10^, ^11^ Differences between age groups within countries have also been reported. Health expectancies such as disability-free life expectancy (DFLE) combine information about quantity and quality of remaining years, and provide an improved indication of different scenarios, including whether: the extra years of life are healthy (compression of morbidity)^12^ or unhealthy (expansion of morbidity);^13^ unhealthy years are increasing, but the proportion of life spent healthy is increasing (relative compression) or decreasing (relative expansion);^14^ or morbidity and disability are increasing but severity of disability is not (dynamic equilibrium).^15^

Health expectancies are important as indicators to monitor population health trends and inequalities internationally, nationally, and regionally.^16^, ^17^ Since 2004, the preferred indicator for the EU is healthy life-years, a DFLE based on self-report global activity limitation indicator.^18^ The UK Office for National Statistics has regularly published time series of DFLE (based on limiting long-standing illness) and healthy life expectancy (HLE; based on self-perceived health [SPH]) at birth and age 65 years. These time series suggest that trends in DFLE and HLE are much less consistent than trends in life expectancy. Comparisons of the periods 2005–07 and 2008–10 suggest that compression of morbidity took place in the UK, with increases in DFLE at birth of 1·4 years for men and 1·3 years for women, compared with life expectancy of 0·9 years for men and 0·6 years for women, although at age 65 years only women's DFLE rose significantly.^19^ However, data for England, comparing 2006–08 and 2009–11, show a continued rise in life expectancy that is larger than the increase in DFLE for men at birth and for both men and women at age 65 years—DFLE at birth for women even shows a slight decrease (0·1 years).^20^

Trends in DFLE and HLE in other countries have been reviewed,^21^ but not all show that extra years of life are healthy or free from disability. Beyond a real increase in morbidity and disability, several reasons exist for the recorded increase in number of years with ill health or disability. First, all health expectancy trends are based on self-report and therefore might be affected by rising expectations of health (and thus a lowered threshold for reporting of ill health or disability). Additionally, questions within countries, such as the UK census question, might change over time, reducing comparability. Second, whether increased years with disability or ill health result from increased cognitive or physical functional limitations is impossible to know since mental ill health is only implicitly included (through its effects on disability or self-reported general health). Finally, all trends in healthy life-years and the between-census values for the UK are based on community-dwelling populations only; people in institutions are only surveyed in the 10-year census.

Our aim was to investigate how health expectancies at age 65 years or older changed between 1991 and 2011, by use of health measures, identical design, and total population (inclusion of institutional residents) available in the Cognitive Function and Ageing Studies (CFAS I and CFAS II). Specifically, we investigated whether (absolute or relative) compression, expansion, or dynamic equilibrium of morbidity took place.

## Methods

### Study design

Data for CFAS I were taken from baseline interviews done between 1989 and 1994 in the population aged 65 years or older in three (Cambridgeshire, Newcastle, and Nottingham) of the six geographical areas of the UK Medical Research Council CFAS. CFAS II baseline interviews were done between 2008 and 2011 in the same three areas and with the same study design and methods as in CFAS I. The sampling base for both studies was primary care registers within the areas, each of which provided 2500 individuals aged 65 years or older, with stratification by age group (65–74 years *vs* ≥75 years; 1250 people per stratum per area). We used oversampling to allow for losses (death, incorrect registration, ineligibility, general practitioner refusals, or participant or gatekeeper refusals). The primary-care practices screened records of patients in selected samples regularly for deaths and terminal illness. Selected individuals were sent an introductory letter from their family doctor; this letter was followed by a visit from a named study interviewer. Full details of the study design, methods, and response rates have been published.^22^

### Health domains

We used three health measures as a basis for calculation of health expectancies: SPH, cognitive impairment, and disability. SPH was measured by asking “would you say that for someone of your age, your health in general is excellent/good/fair/poor”, and participants were categorised as having excellent–good versus fair–poor health. Cognitive impairment was defined by a Mini-Mental State Examination (MMSE)^23^ score (maximum score 30) as: severe impairment (0–17), mild impairment (18–25), or no impairment (26–30).^24^ We used a measure of disability based on basic activities of daily living (BADL) and instrumental activities of daily living (IADL).^25^ Participants were classified as having moderate–severe disability if they were unable to do at least one of five activities without human help: transfer to and from a chair (from interviewer assessment); put on shoes and socks; prepare a hot meal; get around outside; or have a bath or all-over wash. Participants who were able to do all five activities without help from another person but who needed help with at least one of the two additional IADLs (shop, including carrying of heavy bags, and do heavy housework) were classified as having mild disability.

### Statistical analysis

The age-specific and sex-specific prevalence estimates of each health measure were calculated for CFAS I and CFAS II with inverse probability weighting to account for non-response differences between studies and selection of study design. Details of the weighting methods have been published previously.^22^ Analysis of change over time in prevalence of each health measure was done by logistic regression. Models were fitted with time (0=1991, 1=2011), age (5-year age band), and sex, then further adjusted for Townsend deprivation index (in CFAS I tertiles),^26^ education (0–9 years, 10–11 years, or ≥12 years), and region. Because proportional odds assumptions were violated for all three measures, separate models were fitted to any morbidity (fair–poor SPH, MMSE 0–25, or any disability) and severe morbidity (poor SPH, MMSE 0–17, and moderate–severe disability). The proportion of missing data for any of the health measures was low: 2·9% (1991) versus 4·2% (2011) for SPH; 1·8% (1991) versus 3·7% (2011) for MMSE; and 1·1% (1991) versus 4·2% (2011) for disability.

Three main health expectancies—HLE, cognitive-impairment-free life expectancy (CIFLE), and DFLE—were calculated for the combined three regions common to CFAS I and CFAS II by sex for both timepoints (1991 and 2011) by the Sullivan method.^27^ This method applies the age-specific and sex-specific prevalence of the health measure to a standard life table for the same period. To assess dynamic equilibrium, we separated years with fair and poor health, mild and severe cognitive impairment, and mild and severe disability, within each health expectancy calculation.

For the standard abridged life table calculations, we used population mid-year estimates and vital statistics death data provided by the Office of National Statistics^28^ at the district level for the three regions. Instead of using an average a_x_ (the fraction of interval lived by those dying in the interval) of 0·5 in life table calculations, we calculated a precise national a_x_ (and stratified by sex) from national mortality data for both years and used this value in the abridged local life table calculations. We closed life tables at age 90 years.

Prevalence modelling was undertaken in SAS, version 9.3, and all health expectancy calculations were done in R, version 3.0.3.

### Role of the funding source

The funders are represented on the CFAS Management Committee and the Biological Resource Advisory Committee but they had no role in study design, data collection, data analysis, data interpretation, or writing of the report. The corresponding author had full access to all data in the study and had final responsibility for the decision to submit for publication.

## Results

We report changes over time in the components of the health expectancies—ie, life expectancy and the prevalence of each health measure. In the three regions combined between 1991 and 2011, life expectancy rose by 4·5 years for men and 3·6 years for women at age 65 years and by 3·0 years for men and 2·5 years for women at age 70 years; from age 80 years, increases for women exceeded those for men (appendix).

In the three regions combined, 7635 people participated in CFAS I and 7796 in CFAS II. Because of age stratification, proportions of men and women in each age group were similar in 1991 and 2011, but a smaller proportion of women was included in 2011 (55%) than in 1991 (60%) (table 1). Improved access to education (and change in minimum school leaving age by birth cohort) was evident, with more than twice as many participants reporting 12 or more years of education in 2011 as in 1991 (22% *vs* 9%), and decreased deprivation in 2011 than in 1991 (table 1).

Prevalence of cognitive impairment, fair or poor health, and disability was higher in women than in men and increased with age, although less so for SPH than for the other health measures (appendix). After adjustment for age and sex, prevalence of fair or poor SPH (odds ratio [OR] 0·83, 95% CI 0·78–0·88), any cognitive impairment (0·53, 0·49–0·56), severe cognitive impairment (0·49, 0·43–0·56), and moderate–severe disability (0·76, 0·70–0·82) substantially decreased between 1991 and 2011, but that of any disability increased (1·22, 1·14–1·30). These differences remained, but were attenuated after adjustment for region, education, and deprivation. However, the reduction in prevalence of any disability increased further (1·36, 1·27–1·47) after adjustment, and the lowered prevalence of fair–poor SPH was not statistically significant (1·02, 0·89–1·17) after adjustment. The raised prevalence of any disability was not accounted for by changes in cognitive impairment (1·56, 1·44–1·68) or by vision or hearing problems (1·33, 1·21–1·44). However, lessened prevalence of moderate–severe disability was not significant after adjustment for changes in cognitive impairment (0·99, 0·90–1·09).

Between 1991 and 2011, we identified absolute compression of cognitive morbidity at age 65 years for women, with gains in CIFLE of 4·4 years (95% CI 4·3–4·5) and a drop in years with any cognitive impairment (CILE) of 0·7 years (0·2–1·3); the decrease in CILE consisted of a significant decrease in years with mild or moderate–severe cognitive impairment (figure, table 2). Although CIFLE for men aged 65 years rose by 4·2 years (4·2–4·3) and the proportion of life spent cognitive-impairment free increased, we identified no significant decrease in CILE of any severity (figure, table 2).

For SPH, we identified a compression of morbidity for men and women, with significantly enhanced HLE (3·8 years for men [95% CI 3·5–4·1] and 3·1 years for women [2·7–3·4]), although these increases were less than those in life expectancy. Additionally, we identified a significantly increased proportion of healthy remaining life (4·2 percentage points [2·0–6·5] for men and 3·0 percentage points [1·0–4·9] for women; figure, table 3). For disability, we identified dynamic equilibrium, since the proportion of years of disability-free life fell substantially (5·3 percentage points [3·4–7·2] for men and 9·3 percentage points [7·5–11·1] for women), although the increase in number of years with any disability was higher for years with mild disability (1·3 years [1·1–1·6] for men and 2·5 years [2·2–2·8] for women) than for years with moderate or severe disability (0·5 years [0·3–0·8] for men and 0·6 years [0·3–0·9] for women; figure, table 4). Time patterns at age 85 years were similar but with absolute compression of cognitive morbidity and a reduction in the proportion of life spent disability free seen only in women (appendix). CILE and life expectancy with moderate–severe disability are fairly constant with age (figure).

Sensitivity analyses were done for CIFLE, assuming non-responders had twice the risk of severe cognitive impairment as responders, but had no effect on changes in CIFLE over time.

## Discussion

Whether people are living longer, healthier lives than previously and compressing morbidity into a shorter period is a key concern for government, for society as a whole, and for individuals and their families. Our findings show that the answer crucially depends on how health is measured, with absolute compression observed for cognitive morbidity, relative compression observed for SPH, and dynamic equilibrium observed for disability. The increase of 4·4 years for women at age 65 years in CIFLE between 1991 and 2011 was more than the increase in life expectancy (3·6 years), with measurable falls in years with mild (0·5 years [0·3–0·8]) and moderate–severe cognitive impairment (0·2 years [0·1–0·4]), supporting previous findings of a decrease in prevalence of dementia during this period (panel).^22^ Moreover, women spend on average around twice as many years cognitively impaired as do men, and these values are fairly constant with age, as shown by other studies.^35^, ^36^ Findings for disability are less positive than those for cognitive impairment, with a decrease in the proportion of remaining life spent disability free, although the severity of disability seems to be milder than previously.

Cognitive impairment is one reason for difficulty in calculation of IADLs and BADLs, and our findings suggest that reduced moderate–severe disability was a result of decreased cognitive impairment, although increases in any disability were not a result of cognitive impairment. Rises in IADLs and BADLs might be due to increased prevalence of other specific diseases or physical or sensory functional limitations.^37^ Our analyses showed that problems with vision and hearing (self-report or interviewer observed) did not account for rises in disability. By contrast, analysis of health trends from 1992 to 2007 for the population aged 65 years and older from the Health Survey for England showed stability in self-care activities but increased obesity and mobility limitations (walking 200 yards and climbing stairs), which might contribute to gains in mild disability.^3^ This finding accords well with evidence of high prevalence of arthritis in later young-old (aged 65–69 years) cohorts in one centre in CFAS I.^38^

Our study has limitations because of non-response and the subjectivity of two of the measures. Non-response was higher in CFAS II than in CFAS I, as is common in population-based studies. Reasons for non-response did not differ between the studies, although some reasons for refusal (by others on behalf of frail individuals, and by very active individuals) became more prominent.^22^ Because these reasons affect both good and poor health, they are unlikely to substantially bias the overall estimate from CFAS II, as our sensitivity analyses and those of a previous study^22^ showed. Moreover, these reasons would probably result in differences in the same direction for all the health measures, which we did not find. The only difference in study design between CFAS I and CFAS II was the change from two-stage sampling (prevalence screen followed by assessment) in CFAS I to a combined screen and assessment in CFAS II. Since all our health measures are derived from the initial prevalence screen, our findings are not affected by this change in study design or by the attrition that occurred between screen and assessment in CFAS I. Both disability and SPH are self-reported and might be subject to temporal changes in health expectations and thresholds for admission of activity limitation, although self-report of IADLs and BADLs and objective performance measures in very old people (aged 85 years) are consistent in one of the CFAS regions.^39^ Finally, adjustment of health expectancies for education, deprivation, or both needs longitudinal data since life tables are not routinely available for these factors.

One might question what additional information is provided by health expectancy over other measures, since the amount of ill health in a population is often measured by prevalence alone. Nevertheless, as populations age, with more people surviving to the oldest age groups, in which the prevalence of chronic disorders is highest, the overall population prevalence of ill health might increase without individuals being at higher risk of ill health than they were previously. We show this effect by the true age-specific population and mortality rates for women in 1991 and 2011 and a hypothetical prevalence of ill health in 1991 (appendix). Despite a 20% lower prevalence of ill health in each age group in 2011, the overall prevalence in 2011 (9·7%) is higher than that in 1991 (9·3%), and, because of the increased numbers of people at the greatest risk, the absolute number of unhealthy individuals is larger. Health expectancy provides an integrated approach because it takes into account both the changes in living with ill health (prevalence) and changes in mortality that are responsible for increased life expectancy. However, health expectancies are independent of population size. Thus, application of hypothetical prevalence in the example to the true life tables for 1991 and 2011 results in an increase of 3·2 healthy years at age 65 years. Improvement of population health in addition to population ageing results in an increase in the part of life expectancy spent healthy, even with an increase in the overall prevalence of ill health due to more people being at risk. Health expectancy is therefore a potent means to identify interactions between health, ill health, and mortality.

Although years free of ill health defined by all measures have increased during the past two decades, absolute compression was observed only for cognitive impairment, and women in particular spend a smaller proportion of remaining life disability free and more years with mild disability than they did previously. The paradox that women have worse health but better survival than men is dependent on the set of minor health deficits included,^40^ and our future work will investigate which diseases and disorders are responsible for increased mild disability and whether patterns prevail across all regions. Nevertheless, our findings have important implications for government, employers, and individuals, specifically for raising of the state pension age and extension of working life, and for community care services and family carers who predominantly support people with mild–moderate disability to enable them to continue living independently.

## Figures and Tables

**Figure:**
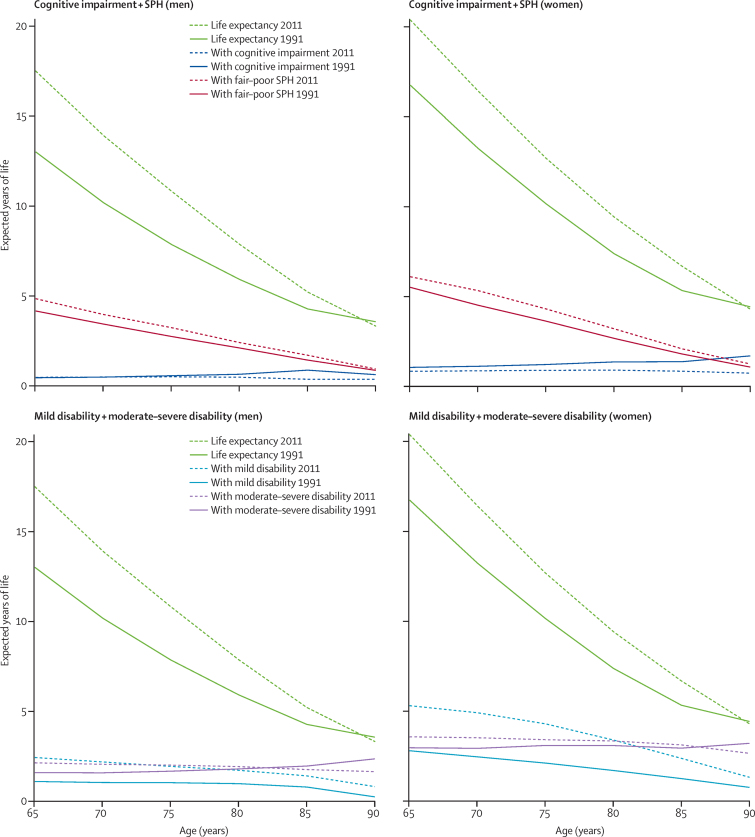
Life expectancy and years lived with cognitive impairment, fair–poor self-perceived health (SPH), mild disability, and moderate–severe disability in 1991 and 2011, all regions combined

**Table 1:** Sociodemographic characteristics of Cognitive Function and Ageing Survey (CFAS) I and CFAS II

	**CFAS I (n=7635)**	**CFAS II (n=7796)**
**Sex**		
Women	4590 (60%)	4246 (55%)
**Age group (years)**		
65–69	1981 (26%)	1939 (25%)
70–74	1776 (23%)	1873 (24%)
75–79	1725 (23%)	1624 (21%)
80–84	1308 (17%)	1290 (17%)
≥85	845 (11%)	1070 (14%)
**Education (years full time)**		
0–9	5529 (74%)	2052 (27%)
10–11	1238 (17%)	3923 (51%)
≥12	692 (9%)	1704 (22%)
Townsend deprivation index (tertile)*		
Low deprivation	2467 (33%)	3737 (48%)
Middle deprivation	2419 (33%)	2412 (31%)
High deprivation	2522 (34%)	1619 (21%)

Data are n (%). Numbers calculated as a percentage of the non-missing values (there were some missing values for education and deprivation).

* CFAS I tertiles.

**Table 2:** Life expectancy, cognitive-impairment-free life expectancy (CIFLE), and proportion of life free of cognitive impairment at age 65 years in 1991 and 2011

	**1991**		**2011**		**Difference (2011–1991)**	
	Men	Women	Men	Women	Men	Women
Life expectancy (years)	13·0	16·7	17·5	20·3	4·5	3·6
CIFLE (MMSE 26–30) (95% CI)	9·4 (9·2 to 9·6)	10·1 (9·8 to 10·4)	13·6 (13·4 to 13·9)	14·5 (14·1 to 14·8)	4·2 (4·2 to 4·3)	4·4 (4·3 to 4·5)
Proportion of life free of cognitive impairment (95% CI)	72·4% (70·6 to 74·3)	60·5% (58·6 to 62·3)	78·2% (76·6 to 79·8)	71·2% (69·5 to 72·9)	5·8% (3·3 to 8·2)	10·7% (8·2 to 13·2)
CILE (MMSE 0–25) (95% CI)	3·6 (3·4 to 3·8)	6·6 (6·4 to 6·8)	3·8 (3·5 to 4·1)	5·9 (5·5 to 6·2)	0·2 (−0·3 to 0·8)	−0·7 (−1·3 to −0·2)
mildCILE (MMSE 18–25) (95% CI)	3·1 (2·7 to 3·6)	5·6 (5·2 to 6·0)	3·4 (2·8 to 3·9)	5·1 (4·5 to 5·6)	0·3 (0·0 to 0·4)	−0·5 (−0·8 to −0·3)
Proportion of life with mild cognitive impairment (95% CI)	24·3% (21·1 to 27·5)	33·5% (31·1 to 36·0)	19·3% (16·3 to 22·3)	25·0% (22·3 to 27·6)	−5·0% (−9·4 to −0·6)	−8·5% (−12·2 to −5·0)
sevCILE (MMSE 0–17) (95% CI)	0·4 (0·3 to 0·5)	1·0 (0·9 to 1·1)	0·4 (0·3 to 0·5)	0·8 (0·7 to 0·9)	0·0 (−0·1 to 0·1)	−0·2 (−0·4 to −0·1)
Proportion of life with severe cognitive impairment (95% CI)	3·2% (−0·3 to 6·8)	6·0% (3·1 to 8·9)	2·5% (−1·0 to 6·1)	3·9% (0·7 to 7·0)	−0·7% (−5·7 to 4·3)	−2·1% (−6·4 to 2·1)

CILE=years with cognitive impairment. mildCILE=years with mild cognitive impairment. sevCILE=years with moderate–severe cognitive impairment.

**Table 3:** Life expectancy, healthy life expectancy (self-perceived health), and proportion of life spent healthy at age 65 years in 1991 and 2011

	**1991**		**2011**		**Difference (2011–1991)**	
	Men	Women	Men	Women	Men	Women
Life expectancy (years)	13·0	16·7	17·5	20·3	4·5	3·6
HLE (95% CI)	8·8 (8·6 to 9·1)	11·2 (11·0 to 11·5)	12·6 (12·4 to 12·9)	14·3 (14·0 to 14·6)	3·8 (3·5 to 4·1)	3·1 (2·7 to 3·4)
Proportion of life spent healthy (95% CI)	68·2% (66·5 to 69·9)	67·3% (65·9 to 68·7)	72·4% (70·9 to 73·9)	70·3% (68·8 to 71·7)	4·2% (2·0 to 6·5)	3·0% (1·0 to 4·9)
unHLE (95% CI)	4·1 (3·9 to 4·3)	5·5 (5·2 to 5·7)	4·8 (4·5 to 5·1)	6·0 (5·8 to 6·3)	0·7 (0·3 to 1·0)	0·5 (0·2 to 1·0)
fairHLE (95% CI)	3·3 (2·8 to 3·7)	4·4 (3·9 to 4·8)	3·7 (3·2 to 4·3)	4·9 (4·4 to 5·5)	0·4 (−0·2 to 1·2)	0·5 (−0·1 to 1·3)
Proportion of life with fair health (95% CI)	25·1% (21·9 to 28·2)	26·2% (23·6 to 28·8)	21·4% (18·4 to 24·4)	24·2% (21·5 to 26·9)	−3·7% (−8·0 to 0·7)	2·0% (−1·8 to 5·7)
poorHLE (95% CI)	0·9 (0·4 to 1·3)	1·1 (0·6 to 1·6)	1·1 (0·5 to 1·6)	1·1 (0·5 to 1·7)	0·2 (0·0 to 0·0)	0·0 (−0·8 to 0·8)
Proportion of life with poor health (95% CI)	6·7% (3·2 to 10·2)	6·5% (3·6 to 9·4)	6·1% (2·8 to 9·4)	5·5% (2·5 to 8·6)	−0·6% (−5·4 to 4·2)	−1·0% (−5·2 to 3·2)

HLE=healthy life expectancy. unHLE=years with fair or poor health. fairHLE=years with fair health. poorHLE=years with poor health.

**Table 4:** Life expectancy, disability-free life expectancy (DFLE), and proportion of life free of disability at age 65 years in 1991 and 2011

	**1991**		**2011**		**Difference (2011–1991)**	
	Men	Women	Men	Women	Men	Women
Life expectancy (years)	13·0	16·7	17·5	20·3	4·5	3·6
DFLE (95% CI)	10·3 (10·2 to 10·5)	11·0 (10·8 to 11·2)	12·9 (12·7 to 13·2)	11·5 (11·3 to 11·8)	2·6 (2·3 to 2·9)	0·5 (0·2 to 0·9)
Proportion of life disability free (95% CI)	79·7% (78·3 to 81·0)	66·1% (64·9 to 67·4)	74·4% (73·0 to 75·8)	56·8% (55·5 to 58·2)	−5·3% (−7·2 to −3·4)	−9·3% (−11·1 to −7·5)
DLE (95% CI)	2·6 (2·5 to 2·8)	5·7 (5·4 to 5·9)	4·5 (4·3 to 4·8)	8·8 (8·5 to 9·0)	1·9 (1·6 to 2·2)	3·1 (2·8 to 3·5)
mildDLE (95% CI)	1·1 (0·9 to 1·2)	2·7 (2·6 to 2·9)	2·4 (2·2 to 2·6)	5·2 (5·0 to 5·6)	1·3 (1·1 to 1·6)	2·5 (2·2 to 2·8)
Proportion of life with mild disability (95% CI)	8·2% (7·3 to 9·2)	16·4% (15·4 to 17·5)	13·8% (12·6 to 15·0)	25·8% (24·5 to 27·2)	5·6% (4·1 to 7·1)	9·4% (7·7 to 11·1)
sevDLE (95% CI)	1·6 (1·4 to 1·7)	2·9 (2·7 to 3·1)	2·1 (1·9 to 2·2)	3·5 (3·2 to 3·7)	0·5 (0·3 to 0·8)	0·6 (0·3 to 0·9)
Proportion of life with moderate–severe disability (95% CI)	12·0% (10·9 to 13·1)	17·4% (16·5 to 18·4)	11·8% (10·7 to 12·9)	17·3% (16·2 to 18·4)	−0·2% (−1·8 to 1·3)	−0·1% (−1·6 to 1·3)

DLE=years with any disability. mildDLE=years with mild disability. sevDLE=years with moderate–severe disability.

## Panel Research in context

**Systematic review**

We updated a previous review of trends in health expectancies^21^ by searching MEDLINE and Web of Science (from Jan 1, 2009, to March 30, 2014) with the search terms “healthy life years”, “free life expectancy”, “active life expectancy”, “healthy life expectancy”, “health expectancy”, and “years of healthy life”. We excluded studies with only one timepoint. We identified no UK studies that included the population in care settings or that reported time differences in cognitive-impairment-free life expectancy.

**Interpretation**

Time differences in health expectancies based on self-rated health and disability are more numerous than those based on cognitive function and are often available nationally. Our findings of compression in fair–poor self-perceived health are in line with others across Europe.^29^, ^30^ Evidence of expansion of mild disability has been reported elsewhere,^31^, ^32^ although some countries have experienced compression of disability.^2^, ^33^ For the UK, estimates of health expectancies for the total population, including people in institutions, are only available from the 10-year censuses, and because the underlying disability (limiting longstanding illness) question changed between 1991 and 2001, our time differences with identical questions and study design provide a more accurate view of change than does the census.

Reduced mortality from coronary heart disease and stroke will have contributed to increased life expectancy, but would probably increase years with cognitive impairment and moderate or severe disability, for which we showed no evidence. However, evidence suggests that prevalence of sensory impairments (hearing and sight) and cognitive impairment, have likewise reduced in the past two decades, together with measured markers of disease such as hypertension, high cholesterol, and C-reactive protein,^3^ all of which, through improved disease management, might have reduced the severity of disability. Nevertheless, increased obesity and mobility limitations, and increased prevalence of arthritis, might have contributed to increases in mild disability. We have previously elucidated the contribution of specific diseases and disorders to disability-free life expectancy.^34^ We need to quantify whether our findings are due to diseases and disorders becoming less disabling during the past two decades.
