# Supplementary material for: A comparison of health expectancies over two decades in England: results of the Cognitive Function and Ageing Study I and II
Source: Lancet. 2016 Feb 20;387(10020):779–86. doi: 10.1016/S0140-6736(15)00947-2 (PMC4761658; doi:10.1016/S0140-6736(15)00947-2)

THE LANCET

Supplementary appendix

This appendix formed part of the original submission and has been peer reviewed. We post it as supplied by the authors.

Supplement to: Jagger C, Matthews FE, Wohland P, et al, on behalf of the Medical Research Council Cognitive Function and Ageing Collaboration. A comparison of health expectancies over two decades in England: results of the Cognitive Function and Ageing Study I and II. *Lancet* 2015; published online Dec 8. http://dx.doi.org/10.1016/S0140-6736(15)00947-2.

Supplementary material

This material formed part of the original submission and has been peer reviewed.

Supplement to: Jagger C, Matthews FE, Wohland P, et al, on behalf of the Medical Research Council Cognitive Function and Ageing Collaboration. A comparison of health expectancies from the Cognitive Function and Ageing Studies over two decades in England. *Lancet* 2015.

Supplementary Table 1: Prevalence of cognitive impairment by severity (no impairment MMSE 26-30, mild impairment MMSE 18-25, severe impairment MMSE 0-17), CFAS I and CFAS II, by age group and sex

	**No cognitive impairment**		**Mild impairment**		**Severe impairment**	
	**CFAS I % (95% CI)**	**CFAS II % (95% CI)**	**CFAS I % (95% CI)**	**CFAS II % (95% CI)**	**CFAS I % (95% CI)**	**CFAS II % (95% CI)**
**Men**						
**65-69 years**	83·1 (80·6 to 85·4)	87·9 (86·2 to 89·5)	16·0 (13·6 to 18·4)	11·5 (9·9 to 13·1)	1·0 (0·4 to 1·6)	0·6 (0·2 to 1·0)
**70-74 years**	78·6 (75·7 to 81·4)	84·3 (82·4 to 86·2)	20·0 (17·3 to 22·8)	14·7 (12·8 to 16·5)	1·4 (0·6 to 2·2)	1·0 (0·5 to 1·6)
**75-79 years**	66·2 (62·8 to 69·7)	76·5 (74·2 to 78·9)	30·3 (26·9 to 33·6)	20·9 (18·6 to 23·1)	3·5 (2·2 to 4·8)	2·6 (1·7 to 3·5)
**80-84 years**	55·2 (50·7 to 59·6)	71·6 (68·7 to 74·5)	39·8 (35·4 to 44·2)	23·3 (20·6 to 26·1)	5·0 (3·1 to 7·0)	5·0 (3·6 to 6·5)
**85+ years**	36·6 (30·3 to 43·0)	56·5 (52·7 to 60·3)	43·2 (36·7 to 49·7)	37·2 (33·5 to 40·9)	20·2 (14·9 to 25·4)	6·3 (4·4 to 8·1)
**Women**						
**65-69 years**	75·6 (73·0 to 78·1)	86·9 (85·3 to 88·6)	23·6 (21·1 to 26·1)	12·7 (11·1 to 14·4)	0·9 (0·3 to 1·4)	0·4 (0·1 to 0·6)
**70-74 years**	70·5 (67·7 to 73·3)	80·2 (78·2 to 82·1)	27·8 (25·1 to 30·5)	18·7 (16·8 to 20·7)	1·7 (0·9 to 2·5)	1·1 (0·6 to 1·6)
**75-79 years**	60·4 (57·5 to 63·4)	72·6 (70·3 to 74·9)	36·1 (33·2 to 38·9)	25·0 (22·8 to 27·3)	3·5 (2·4 to 4·6)	2·3 (1·6 to 3·1)
**80-84 years**	41·8 (38·6 to 45·0)	61·5 (58·8 to 64·1)	46·1 (42·9 to 49·3)	32·6 (30·1 to 35·2)	12·1 (10·0 to 14·2)	5·9 (4·6 to 7·2)
**85+ years**	24·5 (21·3 to 27·7)	46·3 (43·5 to 49·1)	50·8 (47·2 to 54·5)	42·0 (39·2 to 44·7)	24·7 (21·5 to 27·8)	11·7 (9·9 to 13·6)

Supplementary Table 2: Prevalence of self-perceived health (SPH), CFAS I and CFAS II, by age group and sex

	Excellent or good SPH		Fair SPH		Poor SPH	
	CFAS I % (95% CI)	CFAS II % (95% CI)	CFAS I % (95% CI)	CFAS II % (95% CI)	CFAS I % (95% CI)	CFAS II % (95% CI)
**Men**						
**65-69 years**	71·2 (68·3 to 74·1)	74·3 (72·2 to 76·5)	22·0 (19·3 to 24·6)	19·1 (17·1 to 21·0)	6·8 (5·2 to 8·4)	6·6 (5·4 to 7·9)
**70-74 years**	68·0 (64·8 to 71·2)	74·4 (72·1 to 76·7)	26·1 (23·0 to 29·1)	19·3 (17·2 to 21·3)	5·9 (4·3 to 7·6)	6·3 (5·0 to 7·6)
**75-79 years**	65·9 (62·4 to 69·4)	71·1 (68·5 to 73·6)	26·2 (23·0 to 29·5)	23·1 (20·7 to 25·4)	7·9 (5·9 to 9·8)	5·9 (4·6 to 7·2)
**80-84 years**	63·5 (59·1 to 67·8)	71·4 (68·5 to 74·3)	29·2 (25·1 to 33·3)	23·1 (20·3 to 25·8)	7·3 (5·0 to 9·7)	5·5 (4·0 to 7·0)
**85+ years**	66·1 (59·6 to 72·5)	67·6 (64·0 to 71·1)	28·0 (21·9 to 34·1)	26·5 (23·1 to 29·8)	5·9 (2·7 to 9·1)	5·9 (4·2 to 7·7)
**Women**						
**65-69 years**	70·2 (67·5 to 72·9)	78·1 (76·0 to 80·1)	23·2 (20·7 to 25·7)	17·0 (15·1 to 18·8)	6·7 (5·2 to 8·2)	4·9 (3·9 to 6·0)
**70-74 years**	69·1 (66·3 to 71·9)	71·2 (69·0 to 73·5)	25·0 (22·3 to 27·6)	23·5 (21·4 to 25·6)	6·0 (4·5 to 7·4)	5·3 (4·2 to 6·4)
**75-79 years**	65·1 (62·2 to 67·9)	66·3 (63·9 to 68·7)	28·0 (23·0 to 29·5)	28·1 (25·8 to 30·5)	7·0 (5·4 to 8·5)	5·5 (4·4 to 6·7)
**80-84 years**	62·2 (59·0 to 65·4)	63·0 (60·4 to 65·7)	31·1 (28·1 to 34·2)	29·6 (27·1 to 32·1)	6·7 (5·1 to 8·3)	7·3 (5·9 to 8·7)
**85+ years**	66·2 (62·5 to 69·8)	69·1 (66·5 to 71·7)	27·6 (24·2 to 31·1)	26·1 (23·6 to 28·6)	6·2 (4·4 to 8·1)	4·8 (3·6 to 6·0)

Supplementary Table 3: Prevalence of disability, CFAS I and CFAS II, by age group and sex

	No disability		Mild disability		Moderate/severe disability	
	**CFAS I % (95% CI)**	**CFAS II % (95% CI)**	**CFAS I % (95% CI)**	**CFAS II % (95% CI)**	**CFAS I % (95% CI)**	**CFAS II % (95% CI)**
**Men**						
**65-69 years**	88·7 (86·6 to 90·7)	85·3 (83·5 to 87·1)	5·1 (3·7 to 6·6)	9·2 (7·7 to 10·6)	6·2 (4·6 to 7·7)	5·5 (4·4 to 6·7)
**70-74 years**	86·1 (83·7 to 88·5)	81·8 (79·8 to 83·8)	6·2 (4·5 to 7·9)	11·1 (9·5 to 12·8)	7·7 (5·9 to 9·6)	7·0 (5·7 to 8·4)
**75-79 years**	77·5 (74·4 to 80·5)	77·3 (75·0 to 79·7)	9·8 (7·6 to 12·0)	12·4 (10·5 to 14·2)	12·7 (10·3 to 15·2)	10·3 (8·6 to 12·0)
**80-84 years**	63·7 (59·4 to 68·0)	66·4 (63·3 to 69·4)	15·1 (11·9 to 18·3)	17·3 (14·8 to 19·7)	21·2 (17·6 to 24·8)	16·4 (14 to 18·7)
**85+ years**	37·4 (31·1 to 43·8)	40·5 (36·7 to 44·3)	19·1 (14·0 to 24·3)	26·7 (23·2 to 30·1)	43·5 (37·0 to 50·0)	32·8 (29·2 to 36·5)
**Women**						
**65-69 years**	81·6 (79·3 to 83·9)	81·4 (79·5 to 83·3)	11·9 (10·0 to 13·8)	13·6 (11·9 to 15·3)	6·5 (5·0 to 7·9)	5·0 (3·9 to 6·1)
**70-74 years**	79·1 (76·6 to 81·5)	71·3 (69·0 to 73·5)	14·3 (12·2 to 16·4)	20·4 (18·4 to 22·4)	6·6 (5·1 to 8·2)	8·3 (6·9 to 9·7)
**75-79 years**	66·5 (63·7 to 69·3)	58·1 (55·6 to 60·7)	17·7 (15·5 to 20·0)	30·0 (27·6 to 32·3)	15·7 (13·6 to 17·9)	11·9 (10·3 to 13·6)
**80-84 years**	47·0 (43·8 to 50·2)	41·0 (38·3 to 43·7)	22·3 (19·6 to 25·0)	36·4 (33·8 to 39·0)	30·7 (27·7 to 33·6)	22·5 (20·3 to 24·8)
**85+ years**	22·7 (19·7 to 25·8)	19·0 (16·8 to 21·2)	22·9 (19·8 to 25·9)	35·1 (32·5 to 37·8)	54·4 (50·8 to 58·1)	45·8 (43·1 to 48·6)

Supplementary Table 4: Life expectancy, cognitive impairment-free life expectancy (CIFLE), and proportion of life free of cognitive impairment at age 85 in 1991 and 2011 and change between 1991 and 2011, by sex

	1991		2011		Difference (2011-1991)	
	Men	Women	Men	Women	Men	Women
**Life expectancy (years)**	4·2	5·3	5·2	6·6	0·9	1·4
**CIFLE (MMSE 26-30) (95% CI)**	1·5 (1·0 to 2·0)	1·3 (0·9 to 1·6)	2·9 (2·5 to 3·3)	3·0 (2·6 to 3·5)	1·4 (1·2 to 1·6)	1·8 (1·6 to 1·9)
**Proportion of life free of cognitive impairment (95% CI)**	36·2 (24·7 to 47·7)	24·2 (17·2 to 31·1)	56·3 (49·0 to 63·5)	46·0 (39·8 to 52·2)	20·1 (6·5 to 33·7)	21·8 (12·5 to 31·1)
**CILE (MMSE 0-25) (95% CI)**	2·7 (2·4 to 2·9)	4·0 (3·9 to 4·1)	2·3 (2·0 to 2·5)	3·6 (3·4 to 3·8)	-0·4 (-0·9 to 0·1)	-0·4 (-0·7 to -0·1)
**mildCILE (MMSE 18-25) (95% CI)**	1·9 (1·4 to 2·3)	2·7 (2·4 to 3·0)	1·9 (1·5 to 2·4)	2·8 (2·4 to 3·2)	0·1 (-0·1 to 0·3)	0·1 (0·0 to 0·3)
**Proportion of life with mild cognitive impairment (95% CI)**	43·8 (33·2 to 54·4)	50·7 (45·0 to 56·3)	37·4 (29·0 to 45·9)	42·1 (35·9 to 48·4)	-6·4 (-20·0 to 7·2)	-8·5 (-17·0 to -0·1)
**sevCILE (MMSE 0-17) (95% CI)**	0·8 (0·3 to 0·5)	1·3 (1·1 to 1·5)	0·3 (0·3 to 0·5)	0·8 (0·6 to 1·0)	-0·5 (-0·8 to -0·2)	-0·5 (-0·8 to -0·3)
**Proportion of life with severe cognitive impairment (95% CI)**	19·8 (7·0 to 32·5)	25·0 (18·0 to 31·9)	6·4 (-4·8 to 17·6)	11·9 (4·1 to 19·8)	-13·4 (-30·3 to 3·6)	-13·1 (-23·5 to -2·6)

CILE=years with cognitive impairment, mildCILE=years with mild cognitive impairment, sevCILE=years with moderate-severe cognitive impairment

Supplementary Table 5: Life expectancy, healthy life expectancy (self-perceived health), and proportion of life healthy at age 85 in 1991 and 2011 and change between 1991 and 2011, by sex

	1991		2011		Difference (2011-1991)	
	Men	Women	Men	Women	Men	Women
**Life expectancy (years)**	4·2	5·3	5·2	6·6	0·9	1·4
**HLE (95% CI)**	2·8 (2·6 to 3·1)	3·5 (3·3 to 3·7)	3·5 (3.3 to 3.8)	4·6 (4·3 to 4·8)	0·7 (0·3 to 1·1)	1·1 (0·7 to 1·4)
**Proportion of life spent healthy (95% CI)**	67·1 (60·2 to 73·9)	66·9 (63·0 to 70·7)	67·8 (62·8 to 72·8)	69·3 (65·5 to 73·2)	0·8 (-7·7 to 9·2)	2·4 (-3·0 to 7·9)
**unHLE (95% CI)**	1·4 (1.1 to 1.7)	1·7 (1·5 to 1·9)	1·7 (1.4 to 1.9)	2·0 (1·8 to 2·3)	0·3 (-0·1 to 0·7)	0·3 (0·0 to 0·6)
**fairHLE (95% CI)**	1·2 (0·6 to 1·7)	1·4 (1·0 to 1·8)	1·4 (0·9 to 1·8)	1·7 (1·2 to 2·2)	0·2 (-0·5 to 0·9)	0·3 (-0·3 to 0·9)
**Proportion of life with fair health (95% CI)**	27·3 (14·7 to 39·9)	27·0 (19·8 to 34·2)	26·2 (16·9 to 35·5)	25·8 (18·6 to 33·0)	1·1 (-14·5 to 16·7)	1·2 (-9·0 to 11·4)
**poorHLE (95% CI)**	0·2 (-0·4 to 0·8)	0·3 (-0·1 to 0·7)	0·3 (-0·2 to 0·8)	0·3 (-0·2 to 0·9)	0·1 (-0·7 to 0·9)	0·0 (-0·7 to 0·7)
**Proportion of life with poor health (95% CI)**	5·5 (-8·7 to 19·8)	6·0 (-2·1 to 14·2)	6·0 (-4·3 to 16·3)	5·0 (-3·3 to 13·2)	0·5 (-17·1 to 18·0)	-1·1 (-12·7 to 10·5)

HLE=healthy life expectancy, unHLE=years with fair or poor health, fairHLE=years with fair health, poorHLE=years with poor health

Supplementary Table 6: Life expectancy, disability-free life expectancy (DFLE), and proportion of life free of disability at age 85 in 1991 and 2011 and change between 1991 and 2011, by sex

	1991		2011		Difference (2011-1991)	
	Men	Women	Men	Women	Men	Women
Life expectancy (years)	4·2	5·3	5·2	6·6	0·9	1·4
DFLE (95% CI)	1·5 (1·3 to 1·8)	1·2 (1·0 to 1·3)	2·1 (1·8 to 2·3)	1·2 (1·1 to 1·5)	0·5 (0·1 to 0·9)	0·1 (-0·2 to 0·3)
Proportion of life disability free (95% CI)	36·5 (29·7 to 43·3)	22·4 (19·2 to 25·6)	40·1 (34·9 to 45·4)	18·7 (15·7 to 21·8)	3·6 (-5·0 to 12·2)	-3·7 (-8·1 to 0·8)
DLE (95% CI)	2·7 (2·4 to 3·0)	4·1 (3·9 to 4·3)	3·1 (2·8 to 3·4)	5·4 (5·2 to 5·6)	0·4 (0·0 to 0·8)	1·3 (1·0 to 1·6)
mildDLE (95% CI)	0·8 (0·5 to 1·0)	1·2 (1·0 to 1·4)	1·4 (1.1 to 1·6)	2·3 (2·1 to 2·6)	0·6 (0·3 to 1·0)	1·1 (0·8 to 1·4)
Proportion of life with mild disability (95% CI)	17·9 (12·7 to 23·0)	22·6 (19·4 to 25·9)	26·6 (21·8 to 31·4)	35·0 (31·1 to 38·9)	8·7 (1·7 to 15·8)	12·4 (7·3 to 17·5)
sevDLE (95% CI)	1·9 (1·6 to 2.2)	2·9 (2·7 to 3·1)	1·7 (1·5 to 2·0)	3·1 (2·7 to 3·3)	-0·2 (-0·6 to 0·2)	0·2 (-0·2 to 0·5)
Proportion of life with severe disability (95% CI)	45·4 (38·5 to 52·3)	54·8 (51·0 to 58·6)	33·4 (28·3 to 38·5)	46·3 (42·3 to 50·3)	-12·0 (-20·6 to -3·4)	-8·5 (-14·0 to -2·9)

DLE=years with any disability, mildDLE=years with mild disability, sevDLE=years with moderate-severe disability

Supplementary Table 7: Worked example of reduction in age-specific prevalence of ill-health and increase in healthy life expectancy (HLE) but increases in overall prevalence of ill-health

	1991			2011					Life expectancy[3]			HLE[3]		
**Age group**	**Number (N)[1]**	**Prevalence ill-health (%)[2]**	**Number unhealthy (N)**	**Number (N)[1]**	**Prevalence ill-health (%)[2]**	**Number unhealthy (N)**		**Age**	**1991**	**2011**	**change 2011-1991**	**1991**	**2011**	**change 2011-1991**
**65-69**	16847	2	337	15093	1.6	241		**65**	16.7	20.3	3.6	15.0	18.3	3.2
**70-74**	14492	4	580	13353	3.2	427		**70**	13.2	16.4	3.2	11.5	14.3	2.8
**75-79**	12392	8	991	11866	6.4	759		**75**	10.1	12.6	2.5	8.3	10.5	2.2
**80-84**	8705	16	1393	10072	12.8	1289		**80**	7.3	9.4	2.0	5.4	7.3	1.8
**85-89**	4255	32	1362	6524	25.6	1670		**85**	5.3	6.6	1.4	3.3	4.6	1.3
**90+**	1545	50	773	3784	40	1514		**90**	4.4	4.2	-0.1	2.2	2.5	0.4
**TOTAL**	58236	9.3	5435	60692	9.7	5901								

[1]Actual numbers of women in the three CFAS centres in 1991 and 2011
[2]Hypotherical age-specific prevalence of ill-health in 1991 with 2% reduction for 2011.
[3]Life expectancy calculated from true mortality rates in the three CFAS centres in 1991 and 2011. HLE calculated by the Sullivan method as previously using the hypothetical prevalence of ill-health.

Supplementary Figure 1: Life expectancy by age and sex, 1991 and 2011, all centres combined

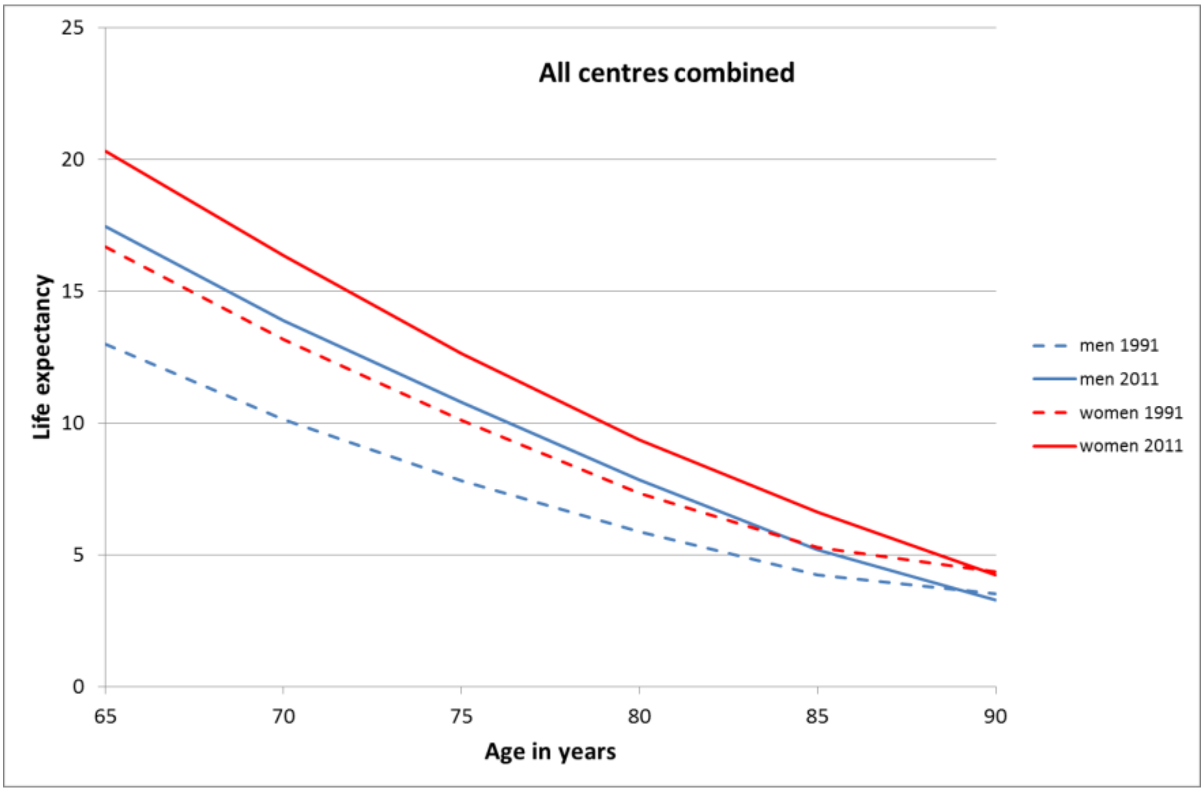

Supplement: Supplementary appendix [file mmc1.pdf]
